# Prevalence and burden of vitiligo in Africa, the Middle East and Latin America

**DOI:** 10.1002/ski2.317

**Published:** 2023-12-18

**Authors:** Anwar Al Hammadi, Caio Cesar Silva de Castro, Nisha V. Parmar, Javier Ubogui, Nael Hatatah, Haytham Mohamed Ahmed, Lyndon Llamado

**Affiliations:** ^1^ Dermamed Clinic Dubai United Arab Emirates; ^2^ Department of Dermatology Pontifícia Universidade Católica do Paraná Curitiba Brazil; ^3^ Department of Dermatology Rashid Hospital Dubai United Arab Emirates; ^4^ Psoriahue Medicina Interdisciplinaria Buenos Aires Argentina; ^5^ King Salman Hospital Riyadh Saudi Arabia; ^6^ Pfizer Dubai United Arab Emirates; ^7^ Pfizer Makati Philippines

## Abstract

Vitiligo is a common chronic autoimmune disorder characterized by skin and hair depigmentation that affects 0.5%–2.0% of the global population. Vitiligo is associated with diminished quality of life (QoL) and psychosocial burden. The burden of vitiligo may vary based on skin tone and cultural differences as well as geographical variations in disease awareness, societal stigma, healthcare systems and treatment options. Data on the burden and management of vitiligo in Africa, the Middle East and Latin America are scarce. Literature searches using terms covering vitiligo in Africa, the Middle East and Latin America were conducted using PubMed to identify relevant publications that focused on disease prevalence and burden, QoL and psychosocial impact and disease management between 2011 and 2021. Most of the reviewed studies were conducted in the Middle East, and most Latin American studies were from Brazil. Most studies involved small patient numbers and may not be generalizable. Reported prevalence of vitiligo ranged from 0.18% to 5.3% in Africa and the Middle East, and from 0.04% to 0.57% in Latin America. In several studies, prevalence was higher among female participants. Generally, non‐segmental vitiligo was the dominant clinical variant identified and the age at onset varied widely across studies. Common comorbidities include autoimmune diseases such as Hashimoto's thyroiditis, alopecia areata and diabetes. Few treatment guidelines exist in these regions, with the exceptions of guidelines published by the Brazilian and Argentinian Societies of Dermatology. There is a clear unmet need for large epidemiological studies with uniform methodology to accurately ascertain the true prevalence of vitiligo in Africa, the Middle East and Latin America. Additional data on vitiligo burden and management in Africa and Latin America are also needed, along with local disease management guidelines that consider genetic variation, psychosocial burden and socioeconomic diversity in all 3 regions.

1

**What's already known about this topic?**

- Vitiligo is a complex autoimmune disease with proven impact on the quality of life and psychosocial well‐being of patients.
- Data on the prevalence, burden and management of vitiligo in Africa, the Middle East and Latin America are scarce.

**What does this study add?**

- This review presents available data on the burden of vitiligo in Africa, the Middle East and Latin America, summarizes treatment guidelines and highlights unmet needs.
- Prevalence of vitiligo in Africa and the Middle East ranged from 0.18% to 5.30% and from 0.04% to 0.57% in Latin America.
- With the exception of guidelines from the Brazilian and Argentinian Societies of Dermatology, few treatment guidelines exist in these regions.

## 2 INTRODUCTION

Vitiligo is a chronic autoimmune disease characterized by skin and hair depigmentation resulting from melanocyte loss.^1^ Vitiligo is a multifactorial systemic disease with a pathogenesis that likely involves genetics, autoimmune responses, oxidative stress, and melanocyte detachment, resulting in melanocyte destruction. Based on international consensus, vitiligo is classified as segmental or non‐segmental.^1^, ^2^ Non‐segmental vitiligo is further divided into subtypes based upon the distribution of skin lesions (i.e., generalized, acral or acrofacial, mucosal, localized, universal and mixed pattern). Rare subtypes are included in an undetermined/unclassified group. Segmental and non‐segmental forms of the disease were originally believed to have distinct underlying pathogenetic mechanisms due to their differing clinical presentations, with somatic mosaicism being favoured for the segmental form.^3^ However, recent evidence suggests an overlapping inflammatory pathogenesis for both forms of the disease.^1^

Global prevalence estimates for vitiligo generally range between 0.2% and 2.0%, with some geographic variation.^4^, ^5^ The disease impacts children, adolescents and adults of both sexes and imparts a significant quality of life (QoL) and psychosocial burden.^1^, ^6^ Vitiligo is more noticeable in individuals with darker skin tones and stigma may be influenced by cultural differences. Additionally, patients may face barriers due to disease awareness, stigma, healthcare systems and limited treatment options specific to their geographic region.^7^ For example, individuals with vitiligo in Brazil are disqualified from certain public professions.^8^ Studies that have reported higher Dermatology Life Quality Index (DLQI) scores in patients with vitiligo in the Middle East compared with those in European countries have suggested that skin colour, cultural stigma and disease education as possible factors to explain the variation.^9^ Overall, data on the burden and management of vitiligo in Africa, the Middle East and Latin America are limited.

In this review, we aim to provide an overview of the burden of vitiligo in Africa, the Middle East and Latin America, including disease prevalence estimates, patient characteristics, genetic components and biomarkers. We review the impacts of vitiligo on patient QoL, including psychosocial impact and comorbidities. Finally, we discuss approaches to disease management and guidelines used in these regions, review the patient perspective and highlight unmet needs in current treatment paradigms.

## 3 METHODS

For the purpose of this narrative review, PubMed searches were performed to identify relevant publications from 1 January 2011, through 22 November 2021. For retrieval of publications relating to the Africa and Middle East (AfME) regions, the search terms ‘Vitiligo’ AND ‘Africa’ OR ‘Middle East’ were used with additional separate searches for vitiligo and the individual countries shown in Supplementary Table S1. For retrieval of publications relating to Latin America, the search terms ‘Vitiligo’ AND ‘Latin America’ OR ‘LatAm’ OR ‘South America’ OR ‘Central America’ were used, with additional separate searches conducted for vitiligo and the individual countries shown in Supplementary Table S1. Titles and abstracts of the identified publications were reviewed manually, and the relevant publications were selected for inclusion in this review.

## 4 BURDEN OF VITILIGO IN AFRICA, THE MIDDLE EAST AND LATIN AMERICA

### 4.1 Prevalence estimates

In AfME, the reported prevalence of vitiligo ranged from 0.18% (in Egypt) to 4.20% (in Tanzania and Saudi Arabia) across several individual studies that included children and/or adults (Table 1).^4^, ^5^, ^10^, ^11^, ^12^, ^14^, ^16^, ^19^, ^20^, ^23^ Relatively few studies evaluated the prevalence of vitiligo in Latin America, with estimates of 0.04%–0.57% reported in studies in Brazil and Mexico (Table 2).^4^, ^29^, ^32^ Studies included relatively few patients and there were substantial differences in the characteristics of the included populations (e.g., age, setting), limiting cross‐study comparisons of vitiligo prevalence across Africa, the Middle East and Latin America.

**TABLE 1:** Epidemiological and clinical aspects of vitiligo: data from Africa and the Middle East.

Reference (country)	Type of study	*N*	Study period	Epidemiology	Demographics
Kruger et al., 2012 (global)^4^	Literature review	–	–	Global: 0.5%–2.0%	–
				Africa/Middle East: <0.2%–1.2%	
Zhang et al., 2016 (global)^5^	Meta‐analysis	103 studies	–	Africa: 0.4%–2.5%	–
Degboe at al., 2017 (Benin)^10^	Retrospective	28,339 patients	1988–2008	0.9%	• Male:female ratio: 1:1
					• Mean duration of lesions: 30.9 months (range, 1 month to 40 years)
					• 52.4% of patients had vitiligo vulgaris
					• Common site: head and neck (69.5%)
					• 0.4% associated with alopecia areata
					• 1.2% had a positive family history
Khater et al., 2021 (Egypt)^11^	Cross‐sectional study	185 primary school children	Not published	3.6%	–
Yamamah et al., 2012 (Egypt)^12^	Community‐based study	2194 children age ≤18 years	2008–2009	0.18%	–
Afkhami‐Ardekani et al., 2014 (Iran)^13^	Cross‐sectional study	1100 patients with type 2 diabetes and 1100 healthy adult volunteers	2011	4.9% in patients with diabetes versus 1.8% in control group	• Male:female ratio:
					• Diabetes group: 1:1.7
					• Control group: 1:1.2
Al‐Refu et al., 2012 (Jordan)^14^	Hospital‐based study	2000 consecutive children	2 years	71 patients with vitiligo age <1 year: 0.45%	• Male:female ratio: 1:0.87
					• Mean age of onset: 6.8 years
				Age 1–5 years: 1%	
					• Mean disease duration: 2.4 months (range, 1 week to 5 months) before presentation to the clinic
				Age 5–12 years: 2.1%	
					• 92.9% of patients had non‐segmental vitiligo
					• Common site: head and neck (35.8% cases)
					• 3% associated with alopecia areata
					• 12.6% had first‐degree relatives with vitiligo
					• 11.2% had first‐degree relatives with diabetes
Ajose et al., 2014 (Nigeria)^15^	Prospective, observational study	102 patients with vitiligo	2004–2009	–	• Male:female ratio: 1:1
					• 60% of patients had non‐segmental vitiligo
					• Prevalence of psycho‐morbidity (anxiety and depression): 59%
Madubuko et al., 2021 (Nigeria)^16^	Retrospective chart review	1600 patients (52 with vitiligo)	December 2014 to December 2019	3.3%	• Male: female ratio: 1:1.1
					• Median (IQR) age of presentation: 29.5 (range, 14.75–49.50) years
					• Mean duration of disease: 18.3 months (±21.5 months)
					• 51.9% of patients had generalized vitiligo
					• Common sites: face (36.5%), upper limbs (34.6%) and lower limbs (32.7%)
Oninla et al., 2016 (Nigeria)^17^	Prospective, observational study	441 patients age 0–19 years at outpatient dermatology clinics	October 2009 to September 2012	5.3%	• Most common in children age >2 years
Alissa et al., 2011 (Saudi Arabia)^18^	Retrospective study	4134 patients	2002–2006	–	• Male:female ratio: 1:1.15
					• Mean age of onset: 17.6 years
					• Vitiligo vulgaris was the most common type (42.3%)
					• 42.8% of patients had positive family history
					• 68.2% developed depigmentation in overexposed areas
Rahamathulla 2019 (Saudi Arabia)^19^	Cross‐sectional, observational study	499 patients	September 2016 to December 2016	4.2%	–
Dlova et al., 2019 (South Africa)^20^	Cross‐sectional, descriptive study	3814 dermatology patients	January 2015 to March 2015	3.09%	–
Kiprono et al., 2013 (Tanzania)^21^	Cross‐sectional study	88 patients age >15 years	2009–2010	‐	• Male:female ratio: 1:1.7
					• Mean age of onset: 33.5 years
					• Mean disease duration: 7.9 years (range, 1 month to 40 years)
					• 55.7% of patients had vitiligo vulgaris
Kiprono et al., 2012 (Tanzania)^22^	Cross‐sectional study	122 patients	–	–	• Male:female ratio: 1:1.8
					• Mean age of onset: 26.2 years (±19.5)
					• 50.8% of patients had vitiligo vulgaris
					• Common site: head and neck (41.8%)
					• 8.8% had positive family history
Mponda et al., 2016 (Tanzania)^23^	Descriptive, hospital‐based, cross‐sectional study	142 patients age ≥55 years	January 2013 to April 2013	4.2%	–
Gonul et al., 2012 (Turkey)^24^	Retrospective study	93 patients	–	–	• Male:female ratio: 1:0.90
					• Median age of onset: 33 years (range, 1–66)
					• Median disease duration: 42 months (range, 1 month to 33 years)
					• 62% of patients had vitiligo vulgaris
					• Common site: face (57.3%)
					• 24.8% had positive family history
					• 47.3% had autoimmune‐ + non‐autoimmune– associated disorders
Topal et al., 2016 (Turkey)^25^	Cross‐sectional, descriptive, prospective study	100 patients	June 2014 to May 2015	–	• Male:female ratio: 1:1.4
					• Mean age of onset: 28.9 years (±14.4)
					• Mean disease duration: 80.7.months (±84.7)
					• 37% of patients had acrofacial vitiligo
					• 46% reported anxiety to be associated with vitiligo
Topal et al., 2016 (Turkey)^26^	Retrospective study	100 patients	June 2013 to May 2014	–	• Male:female ratio: 1:1
					• Mean age of onset: 30.2 years (±16.9)
					• Mean disease duration: 4.9 years (±6.7)
					• 39%, 31% and 26% of patients had focal, acrofacial and generalized vitiligo, respectively
					• 27% had positive family history
					• 45% had comorbid systemic disease
Kalkanli et al., 2013 (Turkey)^27^	Observational study	148 patients	–	–	• Male:female ratio: 1.1:1
					• Mean disease duration: 3.86 years (±4.41)
					• Mean age of onset: 21.18 years (±12.99)
					• 35%, 5%, 4% and 54% had focal, segmental, acrofacial and diffuse vitiligo, respectively

Abbreviation: IQR, interquartile range.

**TABLE 2:** Epidemiological and clinical aspects of vitiligo: data from Latin America.

Reference (country)	Type of study	*N*	Study period	Epidemiology	Demographics
Kruger et al., 2012 (Global)^4^	Literature review	–	–	Global: 0.5%–2%	–
				Mexico/Brazil: 0.21/0.04%	
De Barros et al., 2014 (Brazil)^28^	Retrospective, cross‐sectional study	669 patients with vitiligo	January 2001 to May 2006	–	• Male:female ratio: 1:1.6
					• Mean age of onset: 23.6 years (±17.4)
					• 81.8% of patients had non‐segmental vitiligo
					• Common sites: face (32.6% children/adolescents; 23.3% elderly) and hands (24.0% adults)
					• 36.4% children had segmental vitiligo versus 11.3% adults and 6.7% elderly
De Castro et al., 2018 (Brazil)^29^	Population survey	6048 residences (17,004 inhabitants)	January‐June 2017	0.57% overall	• Male:female ratio: 1:1
				<30 years: 0.34%	
				30–60 years: 0.69%	
				>60 years: 0.85%	
De Sousa Marinho et al., 2013 (Brazil)^30^	Retrospective, observational study	119 children and adolescents with vitiligo	2005–2011	–	• Male:female ratio: 1:1.4
					• Age of onset: 4 months to 14 years
					• 34% of patients had generalized vitiligo; 29% had segmental vitiligo
					• 18.5% had another dermatosis
Martins et al., 2020 (Brazil)^31^	Transverse study	701 patients age <18 years	2006–2014	–	• Male:female ratio: 1:1.67
					• Mean age of onset: 5.9 years
					• Common site: head/neck (44.2%)
					• 16.9% of patients had positive family history
					• 53.8% had generalized vitiligo
Tolentino Junior et al., 2019 (Brazil)^32^	Cross‐sectional, descriptive study	407 patients with autoimmune disease	January‐December 2016	0.132%	–
Morales‐Sanchez et al., 2017 (Mexico)^33^	Cross‐sectional study	150 adults with vitiligo	October 2014 to June 2015	–	• Male:female ratio: 1:2.2
					• Mean age of onset: 31.1 years
					• 26% of patients had positive family history
					• 51.3% had generalized vitiligo

### 4.2 Patient characteristics

Selected patient demographic and disease characteristics from studies in Africa, the Middle East and Latin America are summarized in Tables 1 and 2. In AfME, most studies reported a female predominance.^18^, ^22^, ^25^ A female predominance or similar prevalence between sexes was also observed in 2 studies in Latin America.^28^, ^32^

In terms of clinical presentation, non‐segmental vitiligo (also called ‘generalized vitiligo,’ which was used in some studies) was generally the most common clinical variant in Middle Eastern and African patients^10^, ^14^, ^16^, ^18^, ^22^, ^24^ (e.g., 42.2% of patients with vitiligo at a Saudi Arabian dermatology clinic in a large cross‐sectional study^18^). However, differences in nomenclature/classification across studies (e.g., grouping of different non‐segmental variants or not) makes it difficult to compare the relative frequency of different subtypes by region.^2^ The sun‐exposed areas of the head and neck region were the most frequently involved sites in most studies.^14^, ^18^, ^22^, ^24^, ^28^

The age of onset of vitiligo is not consistent, with disease occurring across age groups from children through older adults (Tables 1 and 2). Higher prevalence peaks were reported in patients in their second, third and fourth decades in studies across Africa (Benin^10^ and Tanzania^22^), the Middle East (Turkey^27^ and Saudi Arabia^18^) and Latin America (Brazil^28^, ^34^). Higher prevalence in older individuals was reported in a large population survey in Brazil.^29^

Vitiligo is among the most common non‐infectious dermatoses in children and adolescents,^11^, ^17^, ^19^, ^35^ and several studies aimed to characterize childhood vitiligo in countries including Jordan,^14^ Egypt^36^ and Brazil.^30^, ^31^ Most paediatric vitiligo cases in these regions occurred in school‐aged children (i.e., 5–10 years of age^14^, ^30^) with a mean disease onset approximately age 6 years.^31^, ^36^ Two studies in Brazil reported a predominance in girls,^30^, ^31^ with later disease onset observed in boys.^30^ However, one study suggested this could be due to selection bias with parents being more likely to report cosmetic‐affecting conditions in girls.^31^ Differences in clinical features (e.g., disease activity, sites affected, clinical form) were reported in childhood versus adult‐onset vitiligo.^31^, ^36^, ^37^ For example, some studies found the face, head and neck to be the most common site of onset in children and adolescents, but arms and forearms were the most common in adults^31^, ^36^; other studies found the prevalence of halo nevi to be higher among children and of leukotrichia to be higher in patients with adult‐onset disease.^37^ Most childhood cases are non‐segmental vitiligo (e.g., >90% in one study from Jordan).^14^, ^36^ However, the segmental subtype is more common among children than adults and has an earlier age of onset.^28^, ^30^, ^34^, ^36^

In Brazil, an association between the prevalence of vitiligo and race/latitude was reported in a large population survey, with higher prevalence observed at lower latitude and in people of non‐European and Amerindian races.^29^ Given the scarcity of data on patterns and prevalence of vitiligo in Latin America, this study highlights the need for further studies.

### 4.3 Genetic components and biomarkers

Familial cases of vitiligo are common, and a study in Brazil^34^ supported an association with family history; however, relatively few Tanzanian^22^ and just over 40% of Saudi Arabian^18^ patients had a positive family history (Tables 1 and 2). Several studies, particularly in the Middle East, aimed to evaluate genetic components and biomarkers. However, most studies were fairly small (40 to 200 patients) pilot studies. The tumour necrosis factor (TNF) pathway has been of particular interest, with genetic mutations reported as risk factors for vitiligo in Egyptian^38^ and Saudi Arabian patients.^39^, ^40^ In addition, elevated serum TNFα levels have been associated with vitiligo in Iraqi^41^ and Iranian patients.^40^ Links between other gene candidates and vitiligo are under investigation.^42^, ^43^, ^44^, ^45^, ^46^, ^47^, ^48^, ^49^, ^50^

### 4.4 Comorbidities, psychosocial effects and QoL

#### 4.4.1 Impact of vitiligo on QoL

Most studies that evaluated QoL in patients with vitiligo from Africa, the Middle East or Latin America used the DLQI^21^, ^33^, ^51^, ^52^, ^53^, ^54^ and/or the Vitiligo‐specific QoL instrument (VitiQoL).^33^, ^51^, ^53^, ^55^, ^56^, ^57^, ^58^ Multiple DLQI translations are available, including Arabic and Portuguese versions.^9^, ^54^, ^59^ An Egyptian Arabic version of the Skindex‐16^60^ is also available. To account for social, religious and cultural aspects unique to some regions, a new patient questionnaire was self‐developed to evaluate the QoL of patients with vitiligo in Saudi Arabia.^61^ Given the clinical characteristics of vitiligo (e.g., lack of physical symptoms/pain), use of a disease‐specific instrument like the VitiQoL is more informative than a more general instrument like the DLQI or Skindex‐16.

Despite availability of multiple adaptations of commonly used QoL scales for use in these regions, data on QoL remain limited. Recent studies in patients from AfME and Latin America are summarized in Tables 3 and 4, respectively. Most included small numbers of patients, and variations in designs/instruments limit generalizability and cross‐study comparisons. Among Brazilian patients, vitiligo was among the skin diseases that most impacted QoL, second to psoriasis.^54^ Moderate QoL impairments were also reported in Tanzanian patients (using the DLQI instrument).^21^ However, small‐to‐no impairments were observed in Saudi Arabian^59^ (using the DLQI instrument) and Mexican^33^ patients (using both the DLQI and VitiQoL instruments).

**TABLE 3:** Quality of life in patients with vitiligo: data from AfME.

Reference (country)	QoL instrument	Type of study	*N*	Study period	Demographics	Key findings
Essa et al., 2018 (Egypt)^60^	Skindex‐16 (Egyptian Arabic)	Instrument validation study	500 dermatology patients; 500 healthy controls	January‐June 2015	• Male:female ratio: 1:1.1^a^	• Vitiligo was associated with less QoL impairment (per Skindex‐16 score) than other dermatologic conditions
					• Mean age: 34.4 years^a^	
Hedayat et al., 2016 (Iran)^55^	VitiQoL (Persian)	Cross‐sectional study	173 patients	April 2013 to September 2014	• Male:female ratio: 1.1:1	• Mean (SD) VitiQoL: 30.5 (14.5; range, 0–60 for Persian version)
						• QoL impairment significantly correlated with disease severity
					• Age: ≥16 years	• Psychiatric problems associated with poorer QoL, although this was not statistically significant
Abdullahi et al., 2021 (Nigeria)^58^	VitiQoL	Cross‐sectional study	77 patients	July 2019 to March 2020	• Male:female ratio: 1:1.4	• Mean VitiQoL: 30.51 ± 15.74
						• Stigma component major contributor to QoL impairment
					• Mean age: 39 years	• Factors associated with greater QoL impairment included female sex, higher socioeconomic class and educational level and lack of family history
Anaba et al., 2020 (Nigeria)^51^	VitiQoL and DLQI	Prospective, cross‐sectional study	29 patients	February 2018 to January 2019	• Male:female ratio: 1:1.1	• By VitiQoL, QoL was impaired in 96.6%: 27.6% mild, 24.1% moderate and 44.8% severe
					• Mean age: 40.1 years	• Stigmatization was main item impacted
						• Good correlation between VitiQoL and DLQI
						• Mean VitiQoL score: 37.4 ± 24.4; DLQI mean score: 5.9 ± 4.2
Al‐Mubarak et al., 2011 (Saudi Arabia)^61^	41‐Item dedicated questionnaire developed	Prospective, cross‐sectional study	260	July‐December 2006	• Male:female ratio: 1.2:1	• Vitiligo had significantly higher impact on QoL in females versus males
					• Mean age: 33 (males) and 31 (females) years	
AlOtaibi et al., 2021 (Saudi Arabia)^59^	DLQI	Cross‐ sectional study	391 patients (29 with vitiligo)	September 2019 to February 2020	• Male:female ratio: 1:10.5^b^	• 79.3% of patients with vitiligo reported small to no effect on QoL
					• Mean age: 33 years^b^	• 6.9% reported large or extremely large effect on QoL
Al‐Shammari et al., 2021 (Saudi Arabia)^9^	DLQI (Arabic)	Cross‐sectional study	253 patients	April‐September 2020	• Male:female ratio: 1:1.9	• Median DLQI score: 4
					• Age ≥16 years; 62.1% <30 years	• Greater QoL impairment in married patients and those with non‐segmental or progressive vitiligo
Bin Saif et al., 2013 (Saudi Arabia)^62^	DLQI and FDLQI	Prospective study	141 patients; 141 family members	––	• Male:female ratio: 1:1	• QoL impairments reported in 91.5% of family members of patients with vitiligo
					• Mean age: 30.3 (patients) and 33.2 (family members) years	
Kiprono et al., 2013 (Tanzania)^21^	DLQI	Cross‐sectional study	88 patients	October 2009 to April 2010	• Male:female ratio: 1:1.7	• Associated with moderate QoL impairment (DLQI mean score: 7.2 ± 4.8)
					• Mean age: 41 years	• 24% of patients with vitiligo reported very large effect on QoL
Fawzy et al 2013 (Egypt)^52^	DLQI and SS	Prospective study	104 patients; 108 matched controls	January 2011 to February 2012	• Male:female ratio: 1:1.97	• Showed significant deterioration in QoL (mean total DLQI score: 9.52)
					• Mean age: 32 years	• 42% of patients with vitiligo reported a very large effect on QoL
						• SS significantly higher in patients than in controls
						• Younger, female, educated patients with darker skin phototypes, visible lesions and a positive family history were at higher risk for reduced QoL

Abbreviations: DLQI, Dermatology Life Quality Index; FDLQI, Family Dermatology Life Quality Index; QoL, quality of life; SS, Stress Score; VitiQoL, Vitiligo‐Specific Health‐Related Quality of Life instrument.

^a^ For the overall population of 500 patients with a dermatologic diagnosis; demographic data for the subset of patients with vitiligo not reported.

^b^ For the overall population of 391 patients attending a dermatology outpatient clinic; demographic data for the subset of patients with vitiligo not reported.

**TABLE 4:** Quality of life in patients with vitiligo: Data from Latin America.

Reference (country)	QoL instrument	Type of study	*N*	Study period	Demographics	Key findings
Catucci Boza et al., 2016 (Brazil)^53^	DLQI, VitiQoL (Brazilian Portuguese) and CDLQI	Cross‐sectional study	117 (93 adults; 24 children)	–	• Male:female ratio: 1:2.1 (adults); 1:1 (children)	• Stigma component was major contributor to QoL impairment
					• Mean age: 45.7 (adults) and 10.0 (children) years	• Women had greater QoL impairment versus men
						• In adults, median DLQI score was 3.0 and median VitiQoL score was 37.0
						• In children, median CDLQI score was 3; higher score was associated with older age
Catucci Boza et al., 2015 (Brazil)^57^	DLQI and VitiQoL (Brazilian Portuguese)	Instrument validation study	74 patients	January‐June 2013	• Male:female ratio: 1:2.1	• Validation of culturally adapted Brazilian Portuguese VitiQoL
					• Mean age: 44.7 years	• Scores correlated with DLQI score and patient assessment of disease severity
Tejada et al., 2011 (Brazil)^54^	DLQI	Cross‐ sectional study	548 patients (16 with vitiligo)	November 2008 to May 2009	• Male:female ratio: 1:2.1^a^	• Among dermatoses, vitiligo was associated with one of the highest DLQI scores (median: 13)
					• Mean age: 43.9 years^a^	
Morales‐Sanchez et al., 2017 (Mexico)^33^	DLQI and VitiQoL (Spanish versions)	Cross‐ sectional study	150 patients	October 2014 to June 2015	• Male:female ratio: 1:2.2	• Vitiligo had minimal impact on QoL
						• Mean (SD) DLQI score was 5.2 (5.4) of maximum score of 30
					• Mean age: 38 years	• Mean (SD) VitiQoL score was 32.1 (22.7) of maximum score of 90

Abbreviations: CDLQI, Childhood Dermatology Life Quality Index; DLQI, Dermatology Life Quality Index; QoL, quality of life; VitiQoL, Vitiligo‐Specific Health‐Related Quality of Life instrument.

^a^ For the overall population of 548 patients attending a dermatology outpatient department; demographic data for the subset of patients with vitiligo not reported.

DLQI and VitiQoL items related to stigmatization were impacted among Nigerian^51^ and Brazilian^53^ patients. Several studies note greater QoL impairment in women versus men^53^, ^58^, ^61^ and in patients with genital lesions.^33^ Patients with comorbid psychiatric disease may be particularly vulnerable to the adverse QoL impact of vitiligo.^53^

#### 4.4.2 Psychosocial impact

A recent global systematic review of psychosocial burden in patients with vitiligo highlighted the prevalence of a variety of psychosocial comorbidities, including high prevalence (>50%) of depression, major depressive disorder, sleep disturbances, avoidance and restriction behaviour, self‐consciousness, emotional impairment, relationship difficulties and cognitive impairment.^6^ However, very few studies have evaluated psychosocial burden in Africa and Latin America.^6^, ^33^

Recent studies evaluating psychosocial impact in patients with vitiligo from Africa, the Middle East and Latin America are summarized in Table 5. These studies highlight the need to evaluate psychosocial comorbidities among patients with vitiligo to form a complete clinical picture and identify patients that may benefit from psychological interventions.^63^

**TABLE 5:** Psychosocial impact of vitiligo: data from Africa, the Middle East and Latin America.

Reference (country)	Instruments	Type of study	*N*	Study period	Demographics	Key findings
Africa/Middle East						
Bidaki et al., 2018 (Iran)^63^	Marlowe‐Crowne social Desirability scale	Cross‐sectional study	150 patients	–	• Male:female ratio: 1:1.9	• Single female patients, those with face and neck lesions, and those with shorter (<5 years) disease duration were at greater risk of experiencing lower social acceptance
					• Mean age: 27.6 years	
Hamidizadeh et al., 2020 (Iran)^64^	BAI, BDI, BHS, GHQ 28	Controlled study	100 patients; 100 controls	–	• Male:female ratio: 1:2.0	• Levels of anxiety and hopelessness were significantly higher in patients versus healthy controls
					• Mean age: 34.5 years	• Women with vitiligo reported higher rates of anxiety and hopelessness than men
Ajose et al., 2014 (Nigeria)^15^	Hospital Anxiety and Depression Scale	Observational study	102 patients with vitiligo; 87 patients with albinism	2004–2009	• Male:female ratio: 1:1	• 59% prevalence of psychiatric comorbidities
					• Mean age: 36 years	• Females had higher anxiety scores versus males
						• Genital involvement was associated with anxiety
						• Vitiligo was associated with greater psychiatric distress versus albinism
Alharbi et al., 2020 (Saudi Arabia)^65^	Modified BDI	Cross‐sectional study	308 patients	2019	• Male:female ratio: 1.5:1	• 54.5% prevalence of depression in patients with vitiligo
					• Mean age: 27 years	• Depression associated with younger age, single status, lower education, shorter disease duration and use of phototherapy
Salman et al., 2016 (Turkey)^66^	Liebowitz Social Anxiety Scale, Hospital Anxiety and Depression Scale; DLQI	Cross‐sectional, controlled study	37 vitiligo patients, 37 acne patients; 74 matched controls	Not published	• Male:female ratio: 1:1.18	• Higher levels of social anxiety, anxiety and depression among patients with vitiligo, similar to those with acne
					• Age >18 years	• Psychosocial morbidity was not correlated with age, sex or disease severity but was correlated with QoL impairment
Latin America						
Morales‐Sanchez et al., 2017 (Mexico)^33^	BAI, BDI, DLQI, VitiQoL	Cross‐sectional study	150 patients	October 2014 to June 2015	• Male:female ratio: 1:2.2	• 34% prevalence of depression
						• 60% prevalence of anxiety
					• Mean age: 38 years	• Moderate correlation between QoL and anxiety or depression

Abbreviations: BAI, Beck Anxiety Inventory; BDI, Beck Depression Inventory; BHS, Beck Hopelessness Scale; DLQI, Dermatology Life Quality Index; GHQ 28, General Health Questionnaire; QoL, quality of life; VitiQoL, Vitiligo‐Specific Health‐Related Quality of Life instrument.

#### 4.4.3 Other comorbidities

Vitiligo commonly co‐occurs with other autoimmune disease.^18^, ^24^, ^26^, ^34^, ^67^, ^68^, ^69^ In particular, high rates of autoimmune thyroid disease were reported in adult and paediatric patients in Brazil, Iran, Saudi Arabia and Turkey^18^, ^26^, ^67^, ^68^, ^69^; however, another Turkish study reported relatively low rates of impaired thyroid function and thyroid autoantibodies.^70^

Co‐occurrence with other dermatologic conditions has been demonstrated.^18^, ^26^, ^71^, ^72^ For example, vitiligo was reported in 1%–5% of Tunisian patients with alopecia areata (AA).^71^ Conversely, AA was reported in 5% of Turkish patients with vitiligo.^26^ A high degree of co‐occurrence between vitiligo and psoriasis was observed among Iranian patients.^72^

Comorbidities reported in patients with vitiligo include diabetes, dyslipidaemia, obesity and hypothyroidism (Saudi Arabia) and hypertension (Iran^73^ and Turkey^26^).

Conclusions regarding associations between vitiligo and comorbid autoimmune, dermatological and other conditions are limited by small sample sizes in most studies, lack of consistent design/assessments across studies, and inability to attribute causality; there is a need for further studies particularly in Latin America.

## 5 DISEASE MANAGEMENT AND TREATMENT

### 5.1 Disease management and treatment guidelines

The goal of vitiligo treatment is to halt the progression of the disease, and stimulate and maintain repigmentation thus avoiding psychosocial disease sequalae.^74^ Combination therapies have proven to be more effective than monotherapy^75^ and include phototherapy with narrow‐band ultraviolet B (NB‐UVB) —either local or full body—together with topical calcineurin inhibitors. Alternative combination therapy consists of excimer laser plus topical calcineurin inhibitors. The recent US Food and Drug Administration (FDA) approval of topical ruxolitinib 1.5% cream for non‐segmental vitiligo in patients aged ≥12 years^76^ highlights a new era in vitiligo treatment as it is the first FDA‐approved topical medication for vitiligo.

Our literature review highlighted that disease management approaches typically involve topical or systemic immunosuppressants, phototherapy and/or surgical techniques.^1^ In a survey among Saudi Arabian dermatologists, most (76%) did not view vitiligo as a purely cosmetic problem and 69% encouraged treatment, most commonly with topical corticosteroids (TCS) and NB‐UVB phototherapy.^77^

Published regional and country‐specific treatment guidelines are generally lacking in Africa, the Middle East and Latin America; there remains a need for national evidence‐based guidelines reflecting unique patient features in the different regions to standardize care and improve patient outcomes.^7^ One notable exception is the 2020 guidelines on the treatment of vitiligo by the Brazilian Society of Dermatology, which provide a consensus on clinical and surgical treatment based on best scientific evidence available to date (Supplementary Table S2).^74^ Guidelines from the Argentinian Society of Dermatology are also available (Supplementary Table S2).^78^ Brazilian, Argentinian and international guidelines define standard treatment as TCS and calcineurin inhibitors for unstable and localized cases and corticosteroid oral mini‐pulse therapy for unstable generalized cases.^1^, ^79^ In the few regional studies that included information about treatment, TCS were commonly used in Brazilian and Tanzanian patients.^22^, ^30^ Brazilian guidelines, published prior to the FDA approval of ruxolitinib in 2022, also highlighted that topical and systemic anti‐Janus kinase agents and anti–interleukin 15 receptor immunobiologicals are in development.^74^

Consistent with frequent use of phototherapy, vitiligo was the most common diagnosis underlying referral to phototherapy among Brazilian dermatology patients.^80^ Phototherapy included NB‐UVB, excimer laser, excimer lamp and psoralen (oral and topical) ultraviolet A (PUVA).^1^, ^74^ Brazilian guidelines recommend NB‐UVB phototherapy as the treatment of choice for repigmentation, noting that excimer laser techniques are not appropriate for patients with extensive affected areas and are associated with higher costs.^74^ Argentinian guidelines recommend the use of NB‐UVB phototherapy as first‐line treatment (Supplementary Table S2) and provide direction on the use of excimer light/laser treatment.^78^ Consistent with typical practice in other regions such as Europe,^1^ surveyed Saudi Arabian dermatologists typically chose NB‐UVB for generalized vitiligo, while excimer laser was most commonly used to treat focal and segmental vitiligo.^81^ In a clinical trial in Saudi Arabia (*N* = 48), 308‐nm excimer laser treatment was effective and improved psychosocial QoL, particularly among female patients.^82^

Brazilian guidelines recommend that surgical modalities be reserved primarily for stable segmental disease and generalized vitiligo, with phototherapy before and after surgical treatment.^74^ Pilot studies involving suction blister epidermal graft and non‐cultured epidermal cell suspension for surgical vitiligo management were recently conducted in Brazil.^79^, ^83^ In the Middle East, transplants of epidermal cell suspension (melanocytes and keratinocytes) are increasingly being used and have been well documented by different groups.^84^, ^85^, ^86^, ^87^

Tailored personalized treatment is required for optimal treatment of vitiligo, and some patients prefer cosmetic camouflage or depigmentation approaches.^1^ However, Brazilian guidelines recommend against several commonly used depigmentation approaches.^74^ Real‐world limitations to achieving appropriate treatment include the availability of phototherapy across centres and insurance coverage only funding a limited number of sessions.

### 5.2 Disease education and awareness

In a 2015 survey of Turkish patients with vitiligo, most had knowledge about their illness and its causes.^25^ However, public misconceptions and attitudes about vitiligo were identified in Saudi Arabian surveys.^18^, ^88^ Misconceptions included vitiligo being of infectious origin and due to lack of hygiene. Few patients (4.3%; *N* = 4134) recognized that loss of pigment cells was a cause of vitiligo.^18^

A survey of Iranian dermatologists indicated that most (66%) were interested in continuing medical education regarding psychodermatology, highlighting the need for collaboration between dermatologists and psychiatrists to improve QoL in patients with dermatological conditions.^89^

### 5.3 The patient's journey

There is substantial variability in the time to seek consultation, with variable disease at presentation within and between studies in these regions (Tables 1 and 2). In most parts of the world, the family physician or paediatrician is the first contact for patients with vitiligo, due to their easy accessibility. Depending on these physicians' experience in managing vitiligo, patients may receive initial treatment or be referred to a dermatologist causing a lag in receiving specialist care due to delays in diagnosis and treatment due to wait times or lack of access to specialist care.^7^ There are currently no data available from Africa, the Middle East nor Latin America pertaining to first contact with physicians accessed by patients with vitiligo.

Low treatment adherence has been reported among Egyptian and Saudi Arabian patients^90^, ^91^ and may present a barrier to disease management. Higher adherence is associated with oral treatment/phototherapy, while adherence is particularly low among patients who view treatment as stigmatizing^90^ and those worried about side effects.^91^ Public misconceptions about vitiligo (e.g., infectious origin) identified in a Saudi Arabian survey^88^ may also impact seeking treatment.

## 6 UNMET NEEDS AND FUTURE DIRECTIONS

### 6.1 Unmet needs

Current data on the epidemiology, QoL/psychosocial impact and management of vitiligo in Latin America are scarce. Most studies come from Brazil and may not be representative of the region as a whole. As such, there is a significant need for local epidemiological studies in different populations. There is also a need for current local disease management guidelines that consider the genetically and socioeconomically diverse patient populations in the different regions.

There is often a long duration of vitiligo before patient presentation at a dermatology clinic.^7^ The recent FDA approval of ruxolitinib cream in adolescents and adults heralds a new era in vitiligo treatment. With many new similar topical and systemic drugs in the pipeline, the future of vitiligo treatment appears to be bright, and dermatologists in the region will soon be better equipped with an armamentarium of treatment options to offer their patients with vitiligo. However, not all patients may have access to treatments due to resource limitations in some regions.^7^

While a number of medical, phototherapy and surgical approaches are under evaluation in clinical trials, there is a need for standardized disease nomenclature and outcomes. It will be important to agree on a set of ‘core outcomes’ to be assessed and reported across clinical trials. One study aimed to develop international consensus on a core outcome set for vitiligo trials and determined that repigmentation, side effects and maintenance of gained repigmentation should be measured in all future trials.^92^ Furthermore, new instruments are being developed to reliably measure disease outcomes. For example, the Vitiligo Extent Score was developed as a physician global assessment for disease extent, with defined categories for extent.^93^

### 6.2 Future prospects in minimizing the burden of vitiligo in Africa, the Middle East and Latin America

As much as possible, dermatologists need to work interdisciplinarily with psychologists or psychiatrists to address the individual problems of each patient. Education of the primary care physician regarding the need for early referral of patients with vitiligo to dermatologists will also be important. In resource‐poor settings such as Africa, the Middle East and Latin America, the primary care physician may be the sole physician in contact with patients. Hence, continuous education regarding basic knowledge and updates in vitiligo management should be periodically imparted to healthcare providers, specifically given the stigma associated with the disease.

There is also a need for patient education programmes and patient support groups to combat the myths and stigmas associated with vitiligo and to encourage patients to visit a dermatologist early in the course of their disease. Public awareness campaigns and education may help revert misconceptions, myths and negative attitudes towards vitiligo.

## 7 LIMITATIONS

This narrative review included a wide literature search to cover multiple aspects of the burden of vitiligo in Africa, the Middle East and Latin America; however, relevant articles may potentially have been excluded because of the chosen search terms. In addition, the absence of systematic review methodology may have resulted in possible bias in the articles chosen.

## 8 CONCLUSIONS

There is a substantial burden of vitiligo in Africa, the Middle East and Latin America, in terms of disease prevalence, QoL and psychosocial impact on patients and their families. Most of the studies reviewed were conducted in the Middle East, and the majority of studies in Latin America were from Brazil. There is an unmet need for large epidemiological studies with uniform methodology to accurately ascertain the true prevalence of vitiligo in Africa, the Middle East and Latin America. There is also a need for additional data on vitiligo burden and management in African and Latin American populations.

## CONFLICT OF INTEREST STATEMENT

Caio Cesar Silva de Castro has served as an advisor to Aché Pharma and Sun Pharma and as a consultant to AbbVie and Pfizer. Haytham Mohamed Ahmed and Lyndon Llamado are employees of Pfizer and may hold stock options or shares of Pfizer.

## AUTHOR CONTRIBUTIONS

**Anwar Al Hammadi**: Conceptualization (equal); Methodology (equal); Writing – original draft (equal); Writing – review & editing (equal). **Caio Cesar Silva de Castro**: Conceptualization (equal); Methodology (equal); Writing – original draft (equal); Writing – review & editing (equal). **Nisha V. Parmar**: Conceptualization (equal); Methodology (equal); Writing – original draft (equal); Writing – review & editing (equal). **Javier Ubogui**: Data curation (equal); Methodology (equal); Writing – original draft (equal); Writing – review & editing (equal). **Nael Hatatah**: Conceptualization (equal); Methodology (equal); Writing – original draft (equal); Writing – review & editing (equal). **Haytham Mohamed Ahmed**: Conceptualization (equal); Methodology (equal); Writing – original draft (equal); Writing – review & editing (equal). **Lyndon Llamado**: Conceptualization (equal); Methodology (equal); Writing – original draft (equal); Writing – review & editing (equal).

## ETHICS STATEMENT

Not applicable.

## Supporting information

Supporting Information S1

## Data Availability

The data that support the findings of this study are available in the article and supplementary material.
