# Supplementary material for: Prevalence and burden of vitiligo in Africa, the Middle East and Latin America
Source: Skin Health Dis. 2023 Dec 18;4(1):e317. doi: 10.1002/ski2.317 (PMC10831562; doi:10.1002/ski2.317)
Supplement: Supplementary file 1 — Supporting Information S1 [file SKI2-4-e317-s001.docx]

**The following Supporting Information relates to the following article:**

**Prevalence and Burden of Vitiligo in Africa, the Middle East and Latin America**

Anwar Al Hammadi,^1^* Caio Cesar Silva de Castro,^2^* Nisha V Parmar,^3^* Javier Ubogui,^4^ Nael Hatatah,^5^ Haytham Mohamed Ahmed,^6^ Lyndon Llamado^7^

*^1^Dermamed Clinic, Dubai, United Arab Emirates; ^2^Department of Dermatology, Pontifícia Universidade Católica do Paraná, Curitiba, Brazil; ^3^Department of Dermatology, Rashid Hospital, Dubai, United Arab Emirates; ^4^Psoriahue Medicina Interdisciplinaria, Buenos Aires, Argentina; ^5^King Salman Hospital, Riyadh, Saudi Arabia; ^6^Pfizer Inc Ltd, Dubai, United Arab Emirates; ^7^Pfizer, Makati, Philippines*

* Authors contributed equally.

**Supplementary Table 1** Countries included in search terms (limited to title/abstract)

| Countries included in Africa and Middle East literature search | Countries included in Latin America literature search |
| --- | --- |
| Afghanistan  Algeria  Bahrain  Democratic Republic of Congo  Djibouti  Dubai  Egypt  Iran  Iraq  Jordan  Kuwait  Lebanon  Libya  Morocco  Oman  Pakistan  Palestine  Qatar  Saudi Arabia  Somalia  South Africa  Sudan  Syria  Tunisia  Turkey  United Arab Emirates  Yemen | Belize  Costa Rica  El Salvador  Guatemala  Honduras  Mexico  Nicaragua  Panama  Argentina  Bolivia  Brazil  Chile  Colombia  Ecuador  French Guiana  Guyana  Paraguay  Peru  Suriname  Uruguay  Venezuela  Cuba  Dominican Republic  Haiti  Puerto Rico |

**Supplementary Table 2** Summary of accepted treatment approaches in Brazil^1^ and Argentina^2^

| **Treatment Guidelines of the Brazilian Society for Dermatology** | | | |
| --- | --- | --- | --- |
| **Disease presentation** | **Surface area** | **Recommended treatment** | **Maintenance** |
| Non-segmental disease | | | |
| Unstable | Localized | TC up to 8 weeks^a^  Followed by TCI^b^  ± phototherapy | TCI twice weekly for 6 months to maintain repigmentation |
|  | Extensive | OMP^c^  ± phototherapy^d^ | TCI twice weekly for 6 months to maintain repigmentation |
| Stable | Localized | TC up to 8 weeks^a^  Followed by TCI^b^  + phototherapy  If no response, consider surgery^e^ | – |
|  | Extensive | Phototherapy^d^  If no response, consider surgery in selected areas^e^ | – |
| Segmental | | | |
| Unstable | Localized | TC up to 8 weeks^a^  Followed by TCI^b^  + phototherapy^d^ | – |
|  | Extensive | OMP^c^  ± TCI twice weekly for 6 months to maintain repigmentation | – |
| Stable |  | TC up to 8 weeks  Followed by TCI^b^  ± phototherapy^d^  Consider surgical treatment^e^ | – |
| **Treatment Guidelines of the Argentinian Society for Dermatology** | | | |
| First line of treatment | - Topical corticosteroids - Topical calcineurin inhibitors (includes tacrolimus and pimecrolimus) - Narrow band–UVB phototherapy | | |
| Second line of treatment | - UVA phototherapy - Topical vitamin D analogues - Targeted phototherapy - Oral corticosteroids (mini pulses) - Surgical methods | | |
| Additional treatment options | - KUVA (khellin + UVA phototherapy) - Antioxidants - Afamelanotide - Pseudocatalase - Prostaglandin E - FUVA (l-phenylalanine + UVA phototherapy) - Depigmentation | | |

Localized <10% BSA; extensive >10% BSA.

BSA, body surface area; OMP, oral corticoid mini-pulse; TC, topical corticoid; TCI, topical calcineurin inhibitor (tacrolimus or pimecrolimus). ±, associated or not. ^a^ With clinical monitoring to evaluate local side effects, especially if in genital, facial or skinfold areas. Most reviewers agree that, if the use of TC is necessary, preference should be given to those with low and medium potency in genital, skinfold and facial areas. The use of high- and very-high-potency corticosteroids should be restricted to other body areas. Also, in sensitive areas, the use of TCI can be prioritized to minimize the side effects of topical corticosteroids. ^b^ Tacrolimus (or pimecrolimus) twice daily. ^c^ In children and the elderly, evaluate the risk-benefit of using oral corticosteroid therapy, mainly due to the association between its use and growth deficit and increased fracture risk, in addition to comorbidities that can be triggered or aggravated by the medication use. ^d^ Preferably narrow band–UVB, excimer laser or excimer light. Due to slow responders, a treatment lasting ≥6 months (2-3 weekly sessions) is suggested. In localized cases, give preference to phototherapy treatment that allows irradiation restricted to the lesion area. The association with oral antioxidants, TCI and TC during treatment can be considered. There was a consensus among the reviewers that the genital area should not be irradiated. ^e^ In the absence of Köbner phenomenon.

**References**

1. Dellatorre G, Antelo DAP, Bedrikow RB, et al. Consensus on the treatment of vitiligo—Brazilian Society of Dermatology. *An Bras Dermatol* 2020; **95 Suppl 1**:70-82. doi: 10.1016/j.abd.2020.05.007.
2. Sociedad Argentina de Dermatologia. Consenso sobre vitiligo. Available at: https://sad.org.ar/wp-content/uploads/2019/10/Consenso-vitiligo-2015.pdf (last accessed 2022).
